# miR-544 promotes maturity and antioxidation of stem cell-derived endothelial like cells by regulating the YY1/TET2 signalling axis

**DOI:** 10.1186/s12964-019-0504-6

**Published:** 2020-03-03

**Authors:** Jianming Guo, Qiuling Xiang, Yaojie Xin, Yongyi Huang, Gang Zou, Te Liu

**Affiliations:** 1 Department of Vascular Surgery, Xuanwu Hospital, Capital Medical University, Beijing, 100053 China; 2 Department of Pathology, Yale University School of Medicine, New Haven, 06520 USA; 3 Zhongshan School of Medicine, Sun Yat-sen University, Guangzhou, 510080 China; 4 Department of Otolaryngology, Shuguang Hospital, Shanghai University of Traditional Chinese Medicine, Shanghai, 201203 China; 5 Department of Obstetrics, Shanghai First Maternity and Infant Hospital, Tongji University School of Medicine, Shanghai, 200040 China; 6 Shanghai Geriatric Institute of Chinese Medicine, University of Traditional Chinese Medicine, 365 South Xiangyang Road, Shanghai, 200031 China

**Keywords:** Induced endothelial cell-like cells, Human amniotic epithelial cells, Epigenetics modification, Antioxidation, Cell therapy, Translational medicine

## Abstract

**Background:**

Inflammation and oxidative stress induced by oxidized low density lipoprotein are the main causes of vascular endothelial injury and atherosclerosis. Endothelial cells are important for the formation and repair of blood vessels. However, the detailed mechanism underlying the regulation of maturity and antioxidation of stem cell-derived endothelial like cells remains unclear. Besides, YY1 and TET2 play a key role on epigenetic modifications of proliferation and differentiation of stem cells. However, the regulatory mechanism of epigenetic modification induced by YY1 and TET2 on stem cells to iECICs is also not clear.

**Aim:**

Here, we want to investigate detailed mechanism underlying the regulation of maturity and antioxidation of stem cell-derived iECICs by by YY1 and TET2.

**Methods:**

The qPCR, Western blot, immunohistochemical staining and flow cytometric analysis were used to analyze the expression level of each gene. Luciferase reporter assay was used to detect the binding sites between microRNA and target genes. The hMeDIP-sequence, ChIP-PCR and dot blot were used to detect the 5-hydroxymethylcytosine modification of genomic DNA. ATP, ROS, SOD assay were used to evaluate of oxidative stress in cells. The iECICs transplantation group The ApoE−/− mice were intravenous injected of iECICs to evaluation of therapeutic effect in vivo.

**Results:**

Our studies have found that as the differentiation of human amniotic epithelial cells (HuAECs) is directed towards iECICs in vitro, the expression levels of vascular endothelial cell markers and miR-544 increase significantly and the expression level of YinYang 1 (YY1) decreases significantly. The luciferase reporter assay suggests that Yy1 is one of the targets of miR-544. Hydroxymethylated DNA immunoprecipitation sequencing showed that compared with HuAECs, iECICs had 174 protein-coding DNA sequences with extensive hydroxymethylation modifications. Overexpression of miR-544 inhibits the activity of the YY1/PRC2 complex and promotes the transcription and expression of the ten-eleven translocation 2 (TET2) gene, thereby activating the key factors of the serotonergic synapse pathway, CACNA1F, and CYP2D6. In addition, it promotes ability of maturity, antioxidation and vascular formation in vitro. Meanwhile, transplantation for miR-544-iECICs can significantly relieve oxidative stress injury on ApoE−/− atherosclerotic mice in vivo.

**Conclusions:**

miR-544 regulates the maturity and antioxidation of iECICs derived from HuAECs by regulating the YY1/TET2/serotonergic synapse signalling axis.

Video abstract

## Background

Inflammation and oxidative stress induced by oxidized low density lipoprotein are the main causes of vascular endothelial injury and atherosclerosis [1–6]. Endothelial cells (ECs) are derived from vascular endothelial progenitor cells (EPCs), which are important for the formation and repair of blood vessels [7–9]. In 1997, Asahara et al. isolated CD34+ positive cells from peripheral blood by magnetic activated cell sorting and fluorescence-activated cell sorting and demonstrated their ability to differentiate into mature endothelial cells and form blood vessels in vivo [7]. Subsequently, the presence of EPCs was confirmed in bone marrow, fat, cord blood, and foetal liver [8, 9]. EPCs are involved in both postnatal vasculogenesis and angiogenesis, contributing to regeneration and repair of blood vessels after vascular injury. The discovery of EPCs not only provides important clues for studying the mechanisms of vasculogenesis and angiogenesis but also provides new methods for the treatment of ischemic diseases and the inhibition of cancer metastasis [8, 9]. However, it is difficult to collect enough ECs and culture them in vitro. The in vitro maintenance of progenitor cell characteristics is difficult, which limits its involvement in tissue repair [8, 9]. Besides, the detailed mechanism of the differentiation and maturation of EPCs is still unclear. Human amniotic epithelial cells (HuAECs) are derived from pluripotent epiblast cells of the amniotic cavity [10–13], which highly express embryonic stem cell factors, such as TRA1–60, TRA1–81, SSEA-3/4, OCT-4, SOX-2, and NANOG [10–13]. HuAECs are characterized by self-proliferation and directed differentiation similar to stem cells [10–13]. Besides, HuAECs can differentiate into various tissue cells derived from the three germ layers in a specific induction environment [10–13]. Since the amniotic membrane is considered a medical waste, it is abundantly available, and collecting it for research purposes does not involve ethical issues. Moreover, HuAECs have low immunogenicity, and therefore, are a very promising cell therapy resource [10–13]. Although there have been many reports of induction of directed differentiation of HuAECs, studies in which researchers successfully induced their differentiation into endothelial cell-like cells and revealed the intrinsic mechanisms are rare.

In addition, an increasing number of reports indicates that microRNAs exhibit significant regulatory effects on the development and differentiation of endothelial progenitor cells and the development of cardiovascular diseases [14–17]. MicroRNAs are a class of non-coding RNA molecules between 18 and 27 nucleotides in length [12, 18–21]. MicroRNAs induce silencing of target genes at the post-transcriptional level by complementary binding to a specific site on the 3’UTR of the target gene mRNA [12, 18–21]. Several studies have confirmed that microRNA-induced epigenetic regulation is involved in the development, differentiation, apoptosis, and disease development of eukaryotes [12, 18–21]. It is known that miR-126 maintains the development, regeneration, and integrity of vascular endothelial cells by targeted regulation of the expressions of Spred-1, PIK3R2/p85-β,and VCAM-1 genes; miR-130a regulates the phenotype of vascular endothelial cells that are developing into new blood vessels by targeted regulation of the expressions of GAX and HOXA5; miR-210 promotes the migration of vascular endothelial cells and angiogenesis by the targeted regulation of the expression of Ephrin-A3 and HIF-1α [22]. Paone S et al. reported that vascular endothelial cell-derived extracellular vesicles can promote intercellular signal transductions during the development of atherosclerosis by transferring intracellular microRNAs, thus promoting disease progression [23]. In particular, the study found that miR-133a/b, miR-208a/b, and miR-499 are important diagnostic and/or prognostic markers for different stages of cardiovascular diseases. miR-1 and miR-145b are potential biomarkers of acute coronary syndromes; miR-1 is highly sensitive to acute myocardial infarction; high expression level of miR-145 indicates a poor prognosis for STEMI and acute myocardial infarction [17].

Moreover, many studies have reported that with cell development or the directed differentiation of stem cells, the epigenetic regulations also change dramatically [24–28]. This change is usually accompanied by modifications in DNA hydroxymethylation and histone methylation [24–28]. YY1 and TET2 play a key role in the abovementioned epigenetic modifications. YinYang 1 (YY1) is a widely-distributed transcription factor [29–32]. YY1 contains four Cys-Cys-His-His-type zinc finger motifs that can activate different eukaryotic genes (such as INO80, CREB, c-myc, histone H4, p53, and PARP-1) or inhibit different eukaryotes genes (such as α-actin, IFN-β, and IFN-γ), and regulate the expression of certain viral promoters [29–32]. YY1 can inhibit or activate the transcription of the target promoter by binding to the transcription initiation region repeats; besides, YY1 can also recruit histone acetyltransferase/deacetylase to form a dimer that interacts with DNA to promote the transcriptional regulatory effect of the enhancer-promoter chromatin loop [29–32]. YY1 is generally considered to play an important role in body development and cell differentiation; whereas dysfunction of YY1 leads to transcriptional abnormalities of downstream genes, and therefore, the occurrence of diseases [29–32]. The 5′ methylation of the cytosine residue in DNA is a heritable epigenetic modification that is critical for proper regulation of gene expression, genomic imprinting, and mammalian development. The Ten-Eleven translocation (TET) protein family (TET1, TET2, and TET3) can catalyse the oxidation of methylated cytosine to 5-hydroxymethylcytosine (5-hmC) [33–35]. In addition, TET protein can also oxidize 5-hmC to form 5-formylcytosine (5-fC) and 5-carboxylcytosine (5-caC), both of which are excised by thymidine-DNA glycosylase (TDG). Thus, cytosine oxidation is effectively linked to the base excision repair pathway, suggesting active cytosine demethylation [33–35]. TET2 is the most common mutant gene in myelodysplastic syndrome [33–35]. However, it is unclear whether the 5-hmC modification, induced by TET2, is also involved in the differentiation and maturation of iECICs.

Therefore, based on the above findings, we hypothesized that, with reference to the reported methods of inducing differentiation of induced pluripotent stem (iPS) cells into iECs, it is possible to achieve directed differentiation of HuAECs into iECICs. Besides, the expressions of microRNA, YY1, and TET undergo specific changes, and these factors may form a certain regulatory network, which plays an important role in regulating the maturity and antioxidation of iECICs.

## Materials and methods

A detailed description of all materials and methods can be found in Supplementary Materials and Methods.

### Preparation of HuAECs

Human placentas were obtained with written and informed consent from pregnant woman in Shanghai First Maternity and Infant Hospital, Tongji University School of Medicine. They were negative for HIV-I, hepatitis B, and hepatitis C. Appropriate use of human amnion was approved by the institutional ethics committee. Amnion membranes were mechanically peeled from chorines of placentas obtained from women with an uncomplicated Cesarean section. The epithelial layers with basement membrane attached were obtained and used to harvest HuAECs as previously described, with some modifications [11, 20]. Briefly, the membrane was placed in a 250-mL flask containing DMEM medium and cut with a razor to yield 0.5–1.0 cm^2^ segments. The segments were digested with 0.25% trypsin-EDTA at 37 °C for 45 min. The resulting cell suspensions were seeded in a 6-well plate in DMEM:F12 (1:1) medium supplemented with 15% FBS (PAA, Linz, Austria), 100 μg/ml streptomycin, 100 U/ml penicillin and 0.3 mg/ml glutamine, and incubated in a humidified tissue culture incubator containing 5% CO_2_ at 37 °C.

### Induced differentiation of iEClCs

The protocol of HuAECs inducing to differentiate into iECICs and iECs was as previously described with some modification [36, 37]. Briefly, HuAECs were cultured in mTeSR1 medium (STEMCELL Technologies), changed daily. At day 0, the HuAECs were treated with 6 mM CHIR99021 (Selleckchem) and 10 nM BMP4 (STEMCELL Technologies) for 2 days in DMEM:F12 (1:1) medium which consists of 2.5 mM GlutaMAX and 60 mg/ml ascorbic acid (Sigma-Aldrich, St. Louis, USA). After 2 days, the medium was changed into EGM-2 medium (Lonza) for 3 to 9 additional days.

### The hMeDIP-sequence

Genomic DNA of each group was sonicated to ~ 200–800 bp fragments, and 1 μg of fragmented sample was ligatedto Illumina’s genomic adapters with Genomic DNA Sample Kit (#FC-102-1002, Illumina). DNA samples were repaired as well as a single ‘A’ base was added to the 3′ ends before hMeDIP. ~ 300–900 bp ligated DNA fragments were further immunoprecipitated by anti-5-hydroxymethylcytosine antibody (Diagenode). The enriched DNA was amplified by PCR andpurified by AMPureXP beads. The immunoprecipitated genomic DNA of was purified and sequenced followed bystandard Illumina protocols. Clean reads werealigned to Human genome (UCSC HG19) using HISAT2 software (V2.1.0). Significantlyenriched regions were determined by Model-based Analysis of MACS package. GO term and KEGG pathway analyses were performed by the databasefor annotation, visualization and integrated discovery programs.

### Animal grouping and cell transplantation

A total of 24 specific pathogen-free (SPF)-grade ApoE^−/−^ C57/BALB mice were purchased from the Shanghai Research Center for Model Organisms (license number: SCXK (Shanghai) 2014–0002). The mice were 6–8 weeks of age and weighed 30 ± 5 g. After one week of adaptive feeding with an ordinary diet, the ApoE^−/−^ C57/BABL mice were randomly divided into 4 groups: blank control (mice fed a standard diet), saline model control (mice receiving a high-fat dietand an equal volume of normal saline), miR-544-iECICs transplantation group (mice given the high-fat diet and intravenous injection of GFP-labeled miR-544-iECICs from caudal vein) and miR-mut-iECICs transplantation group (mice given the high-fat diet and intravenous injection of GFP-labeled miR-mut-iECICs from caudal vein). Each group contained 6 mice and was fed for 4 weeks. Cells were injected once a week and 100 μl (1 × 10^8^ cells/ml) were injected at a time. All the animal experiments were conducted in accordance with the guidelines of the NIH for the care and use of laboratory animals. The study protocol was also approved by the Committee on the Use of Live Animals in Teaching and Research, Shanghai University of Traditional Chinese Medicine, Shanghai, China.

### Statistical analysis

Each experiment was performed as least thrice, and data were shown as the mean-standard error where applicable; differences were evaluated with Student’s t-test. A *P* value less than 0.05 was considered statistically significant.

## Results

### The expression level of endogenous Yy1 decreased significantly during differentiation of HuAECs into iECICs

**Fig. 1:**
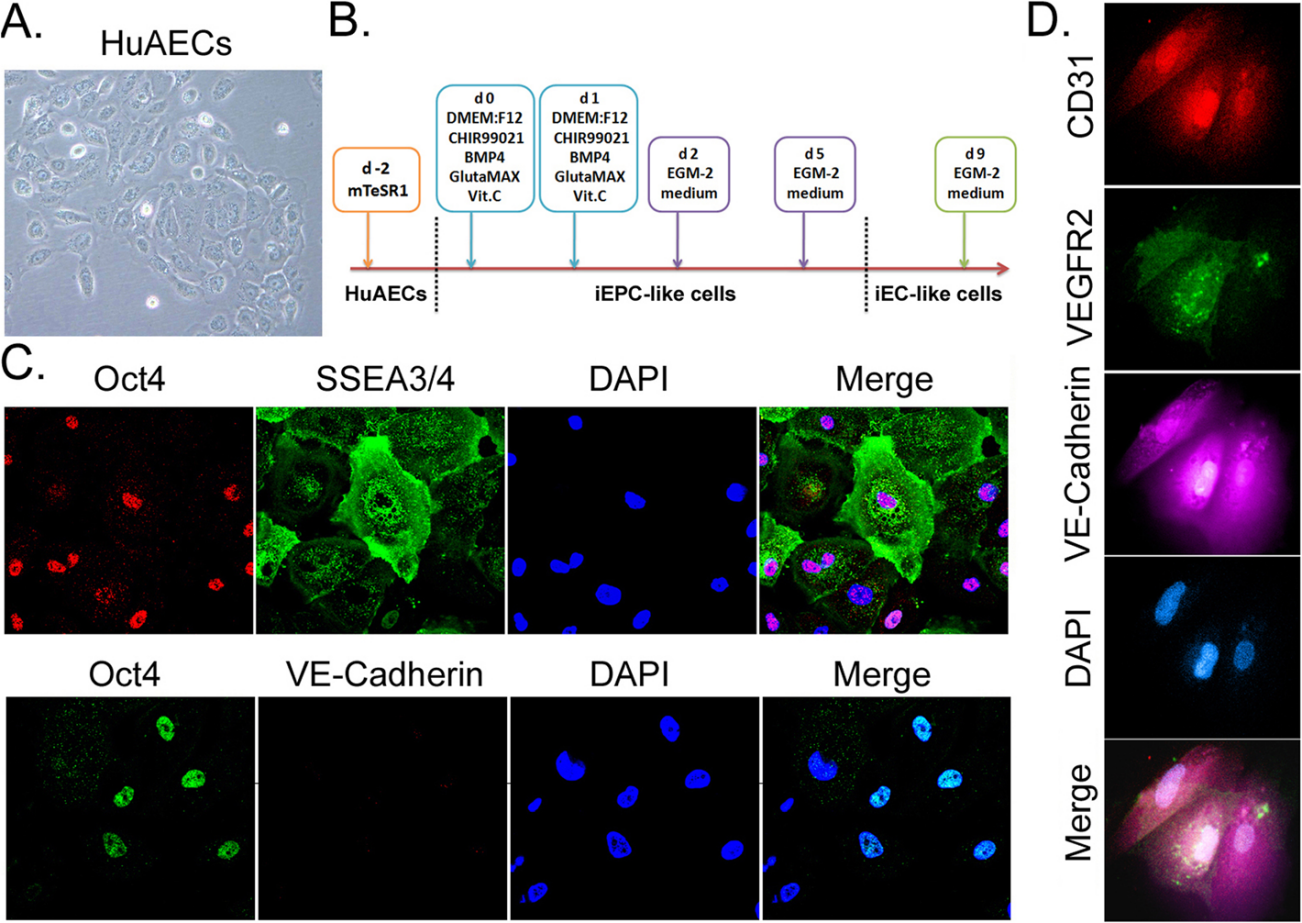
Stepwise induction of differentiation of HuAECs into iEClCs. **a** Phenotype of HuAECs. Magnification: 200×. **b** The flowchart of stepwise induction method. **c** Immunofluorescence staining of HuAECs expressing markers of embryonic stem cells and endothelial cells. Magnification: 200×. **d** Immunofluorescence staining of iEClCs expressing markers of endothelial cells. Magnification: 200 ×

**Fig. 2:**
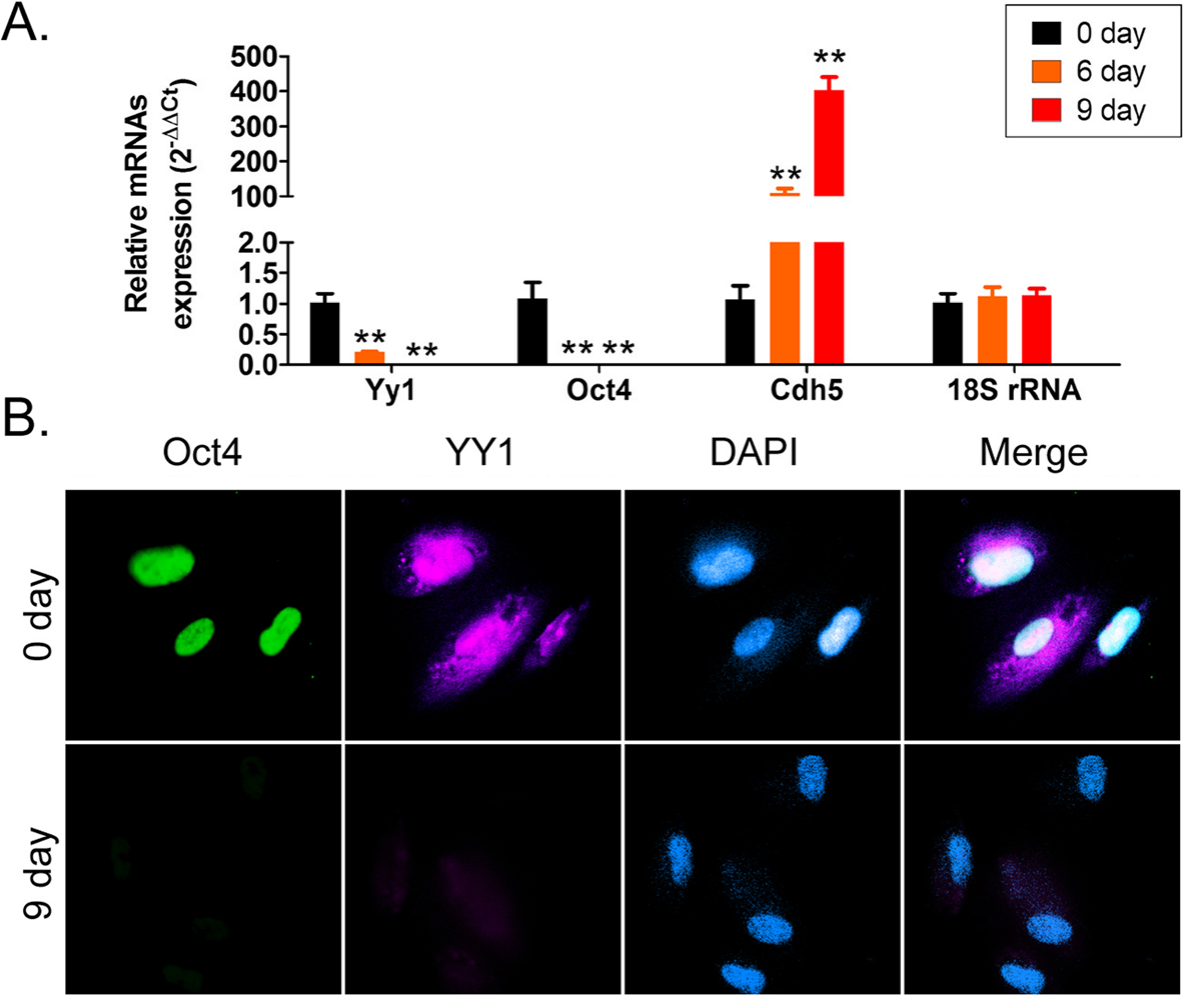
Expression of YY1 at different time points of induction. **a** The results of qPCR assay. **p < 0.01 vs. day0;t test;*n* = 3. **b** Results of immunofluorescence staining of cells before and after induction. Magnification: 200 ×

Nine days after inducing differentiation of HuAECs by the stepwise induction method, immunofluorescence (IF) assay showed that uninduced HuAECs highly expressed the embryonic stem cell markers, such as Oct4, SSEA3/4, etc. (Fig. 1). The induced iECICs highly expressed the endothelial cell markers, such as CD31, VE-Cadherin, and VEGFR2 (Fig. 1). The results showed successful induction of differentiation of HuAECs into iECICs in vitro by stepwise induction method. Subsequently, qPCR results showed that the mRNA expression levels of Oct4 and Yy1 (Yinyang 1) showed a significant decreasing trend at different time points (day 0, day 6, and day 9) during the differentiation of HuAECs into iECICs (Fig. 2). The mRNA expression level of vascular endothelial cell marker, CDH5 (VE-Cadherin), showed an opposite trend to that of the above genes (Fig. 2). The IF results also showed that at day 0, HuAECs highly expressed the Oct4 and YY1 proteins; after 9 days of induction, the expression of YY1 protein was significantly decreased, while the expression of VE-Cadherin protein was significantly increased (Fig. 2). It suggested that the expression of YY1 protein is negatively correlated with the differentiation of vascular endothelial cells.

### Yy1 is a potential regulatory target of miR-544

**Fig. 3:**
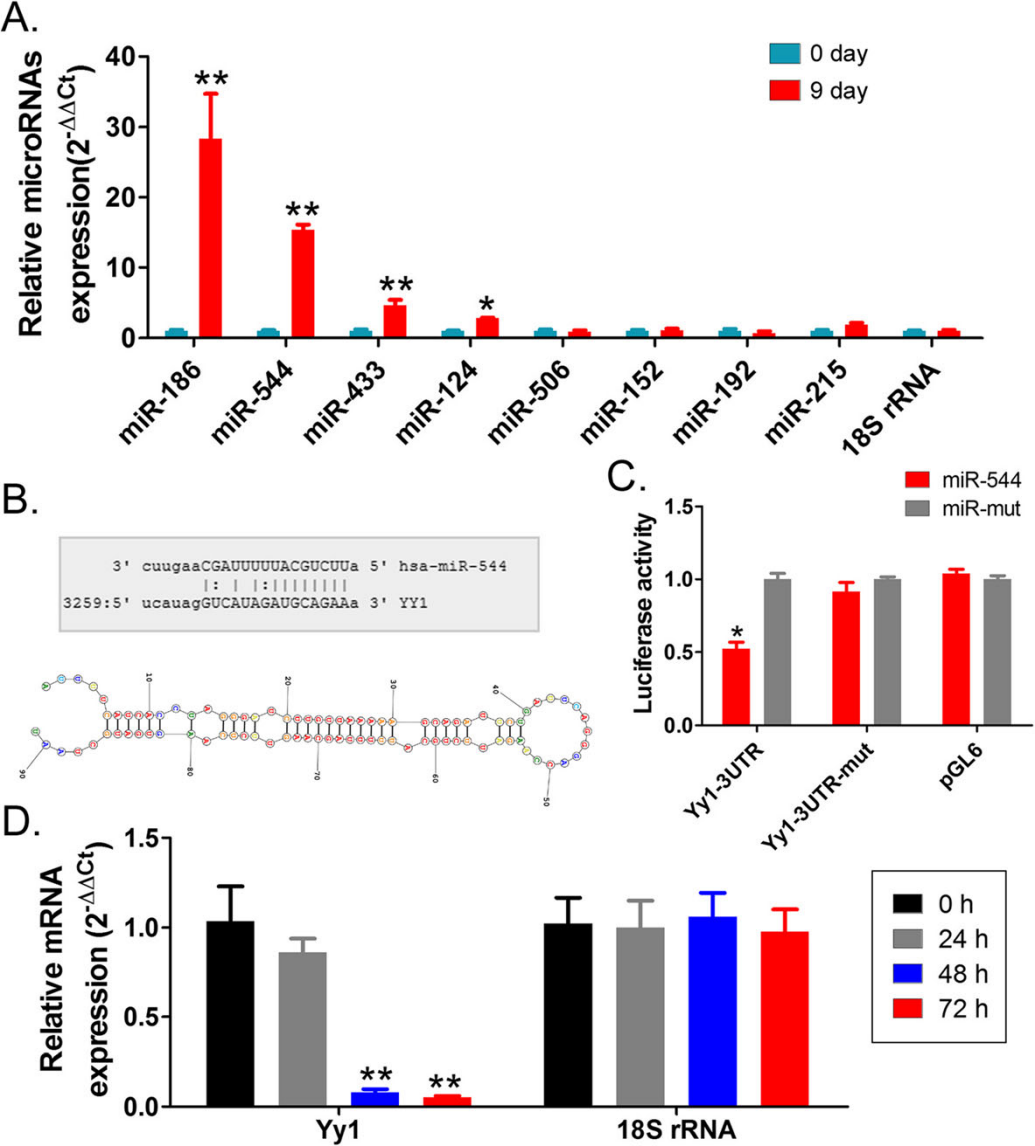
miR-544 targeted silencing of the expression of YY1. **a** The results of qPCR assay of microRNA. **p < 0.01 vs. HuAECs; *p < 0.05 vs. HuAECs; t test; *n* = 3. **b** The results of bioinformatics prediction. **c** The results of luciferase reporter gene assay to identify the targeted binding of miR-544 to the 3′ UTR of Yy1 mRNA. *p < 0.05 vs. miR-mut; t test; *n* = 3. **d** The results of qPCR assay of the expression level of Yy1 mRNA at different time points of induction after overexpression of miR-544. **p < 0.01 vs. 0 h; t test; *n* = 3

We hypothesized that downregulation of Yy1 expression is associated with the regulatory effect of microRNAs. microRNA-qPCR examined eight potential microRNAs that can perform the targeted regulation of Yy1 expression. The results indicated that, among these microRNAs, the expression levels of miR-186, miR-544, miR-433, and miR-124 were significantly higher in the iECICs phase than in the HuAECs phase (Fig. 3). Subsequently, bioinformatics analysis (http://www.microrna.org/microrna/home.do) showed that there was a complete complementary base pairing between the eight nucleotides of mature miR-544 and the 3’UTR (3272 nt - 3279 nt) of Yy1 mRNA (Fig. 3). The luciferase reporter gene assay showed that the activity of luciferase was significantly lower than that of the control group when both miR-544 and the luciferase-encoding plasmid carrying Yy1–3’UTR were present in the cells (Fig. 3). The results suggested that miR-544 induced gene silencing by base pairing with the specific site on the 3’UTR of Yy1 mRNA. Subsequently, after overexpression of exogenous miR-544 oligo microRNA in HuAECs, qPCR results indicated that the expression level of endogenous Yy1 was significantly decreased at 48 h and 72 h after transfection (Fig. 3). It suggested that the expression level of endogenous miR-544 was gradually increased during cell differentiation, which leads to the downregulation of Yy1 expression.

### Overexpression of miR-544 promotes differentiation and maturation of HuAECs-derived iECICs

**Fig. 4:**
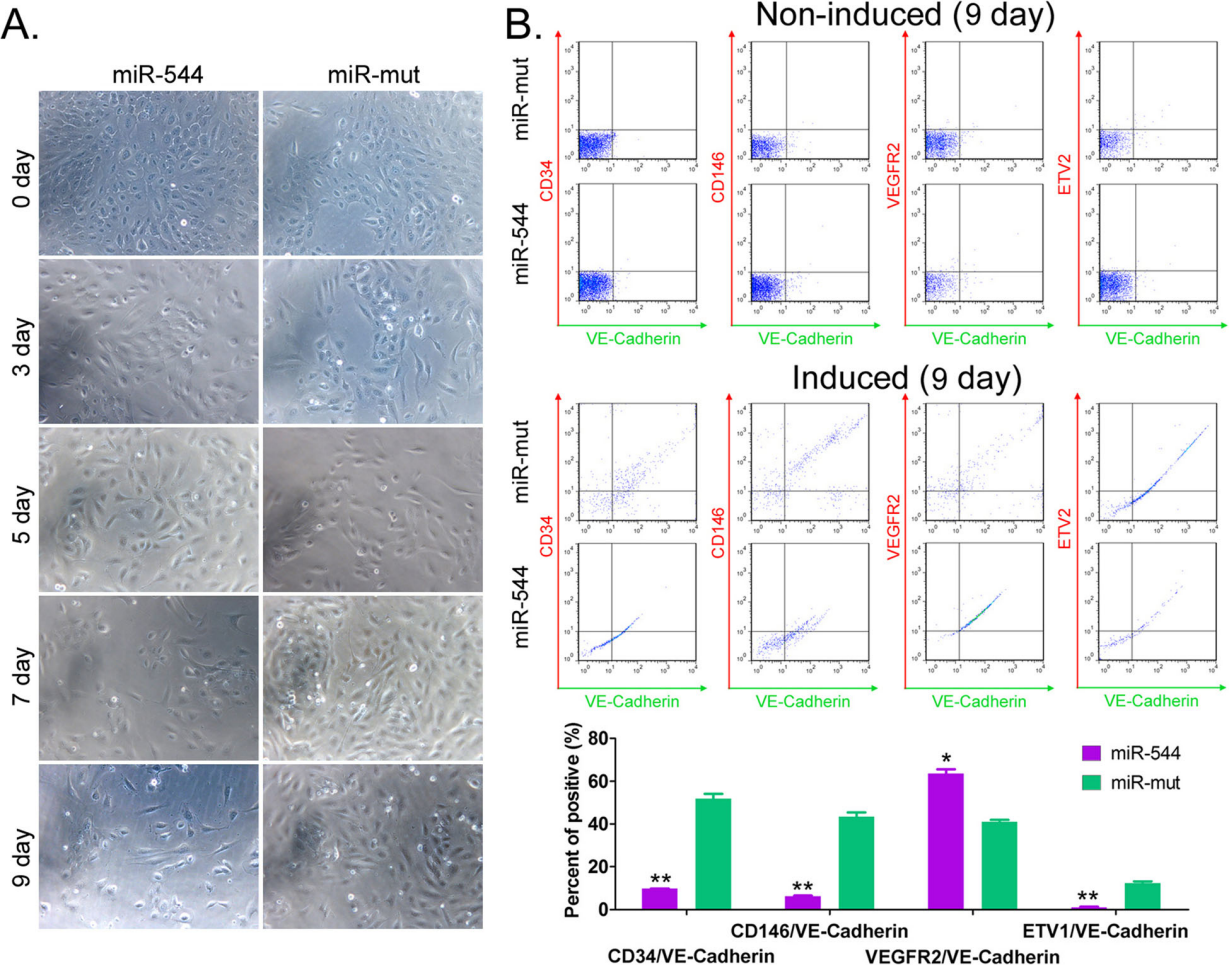
Overexpression of miR-544 promotes differentiation and maturation of endothelial-like cells. **a** The cellular morphology at different time points during the differentiation of HuAECs into iEClCs. Magnification: 200×. **b** Flow cytometry assay of markers of endothelial (progenitor) cells. **p < 0.01 vs. miR-544; t test; n = 3

To investigate the role of miR-544 in inducing the differentiation of HuAECs into iECICs, we overexpressed exogenous miR-544 in HuAECs. At the initial stage of induction (between days 0 and 3), most of the cells of miR-544-HuAECs and miR-mut-HuAECs groups were epithelioid cells with large nuclei, a paving stone arrangement, and strong stereoscopic impression (Fig. 4). However, during later stages, the number of long spindle-shaped endothelial-like cells in the miR-544-HuAECs group increased significantly (Fig. 4). The flow cytometry (FCM) assay revealed that no group of cells expressed markers of vascular endothelial cells (CD34, CD146, VEGFR2, ETV2, VE-Cadherin, etc.) before induction (day 0). On the ninth day of induction, the proportion of double-positive cells, such as CD34+/VE-Cadherin+, CD146+/VE-Cadherin+, and ETV2+/VE-Cadherin+ cells was significantly lower in miR-544-iECICs group than in miR-mut-iECICs group (Fig. 4). However, in miR-544-iECICs group, the proportion of VEGFR2+/VE-Cadherin+ double-positive cells was significantly higher than that in miR-mut-iECICs group (Fig. 4). Considering that proteins such as CD34, CD146+, and ETV2 are markers of endothelial progenitor cells, and VEGFR2 is highly expressed in mature vascular endothelial cells, the above results suggested that overexpression of miR-544 promotes differentiation and maturation of iECICs.

### Overexpression of miR-544 promotes activation of TET2 transcription

**Fig. 5:**
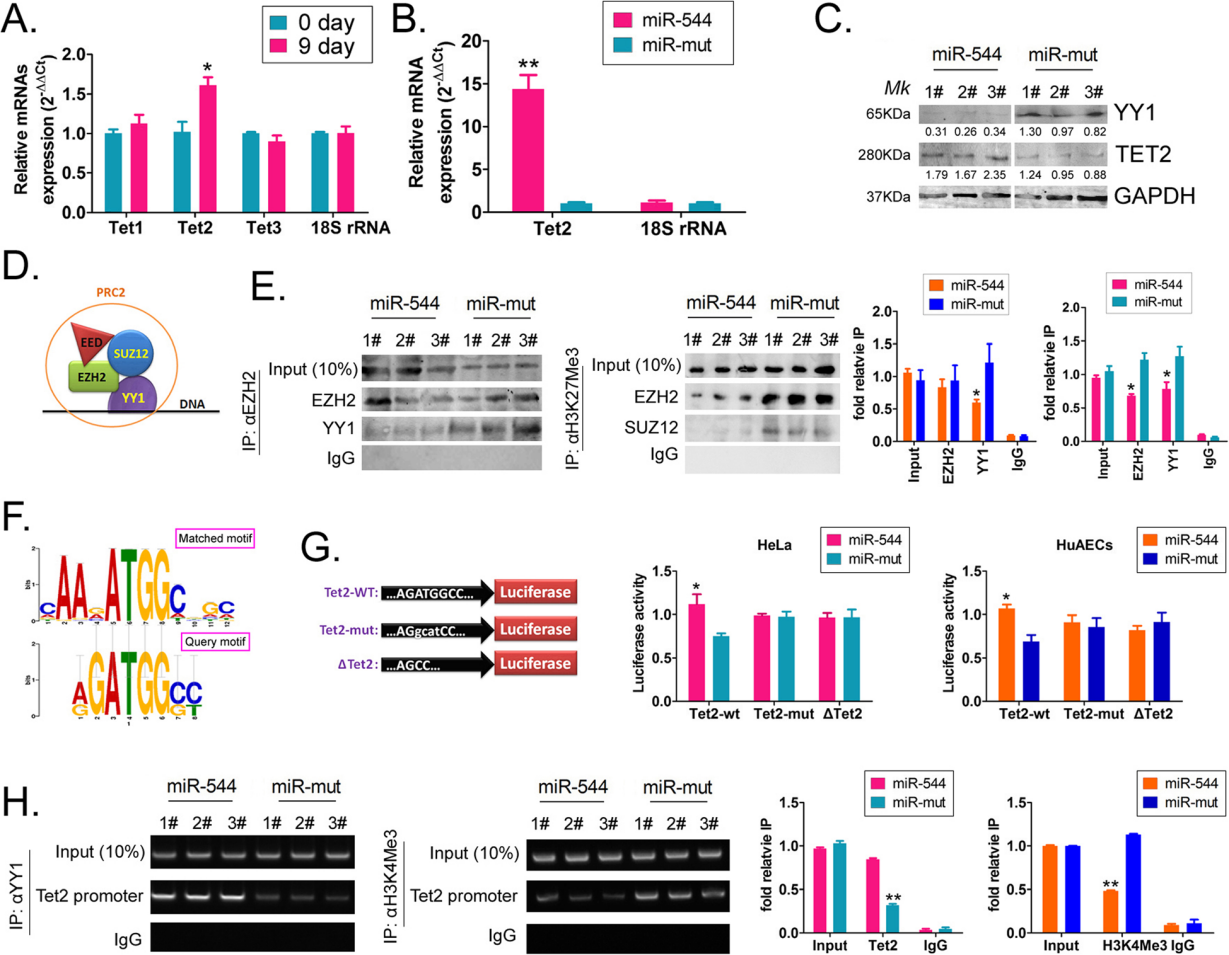
Overexpression of miR-544 promotes TET2 transcription. **a** qPCR analysis of mRNA levels of TET genes before and after the induced differentiation of iEClCs. *p < 0.05 vs. HuAECs; t test; n = 3; **b** qPCR analysis of the expression level of endogenous TET2 mRNA after overexpression of miR-544. **p < 0.01 vs. miR-mut;t test;n = 3. **c** Western blot analysis of the expression level of TET2 in each group of cells. **d** The structure of PRC2. **e** Co-IP western blot analysis of the expression level of PRC2. *p < 0.05 vs. miR-mut; t test; n = 3. (F) The bioinformatics prediction of DNA motif sequence recognizable by YY1. **g** Thestructure of luciferase reporter plasmid and the results of the luciferase reporter gene assay. *p < 0.05 vs. miR-mut; t test; *n* = 3. (H) ChIP-PCR assay of YY1 binding to the promoter of TET2 gene. **p < 0.01 vs. miR-mut;t test;*n* = 3

First, the qPCR results indicated that the expression level of TET2 was significantly increased during the differentiation of HuAECs into iECICs, while the expression levels of TET1 and TET3 were not significantly changed (Fig. 5). When miR-544 was overexpressed in HuAECs, the mRNA level of endogenous TET2 was significantly higher than that of the control group (overexpressing miR-mut) (Fig. 5). In addition, western blot analysis also showed that after overexpression of miR-544 in HuAECs, the level of YY1 protein was significantly lower than that of the control group, and the level of TET2 protein was significantly higher than that of the control group (Fig. 5).Previous studies have indicated that the YY1 protein is a subunit of the polycomb repressive complex 2 (PRC2) (Fig. 5), which is also known to harbour other proteins, such as SUZ12, EZH2, and EED [30–32, 38–40]. Moreover, the complex can induce methylation, demethylation, and trimethylation of Lys27 on histone H3, which is an epigenetic modification marker associated with transcriptional repression [30–32, 38–40]. Therefore, co-immunoprecipitation (co-IP)-western blot analysis showed that, in the miR-544-HuAECs group, the expression of YY1 protein, bound to EZH2, was significantly decreased, and the level of EZH2 protein itself was also significantly decreased, indicating a positive correlation between them (Fig. 5). In addition, in the miR-544-HuAECs, the expression of EZH2 and SUZ12, bound to H3K27Me3, was significantly decreased (Fig. 5). It is predicted by bioinformatics software that YY1 can recognize a specific motif “AGTGGC” of the gene promoter. Meanwhile, the software also predicted that there was a recognition site for YY1 near the − 270 bp of the promoter region of the TET2 gene. Therefore, in order to verify whether YY1 specifically binds to the TET2 promoter and exerts transcriptional repression, we constructed luciferase plasmids carrying different YY1 recognition sequences in the promoter region. Different plasmids were transfected into HeLa cells (considering the high expression of endogenous YY1 in HeLa cells, they were chosen as a positive control) and HuAECs. The luciferase reporter gene assay revealed that when miR-544 or miR-mut was expressed in the cell in the presence of the wild type (WT) TET2-Luciferase plasmid containing the motif sequence recognizable by YY1, the activity of the luciferase in the miR-544 group was significantly higher, and that in the miR-mut group was significantly lower (Fig. 5). However, when miR-544 or miR-mut was expressed in the presence of the TET2-Luciferase plasmid with a mutant motif sequence, there was no significant difference in the luciferase activity between the miR-544 and miR-mut groups (Fig. 5). Therefore, these results confirmed that YY1 negatively regulated the transcriptional activity of TET2 by targeting a specific motif of the promoter. Finally, the ChIP-PCR method further validated the above speculation. A pull-down assay was performed on the chromatin of cells from the miR-544 and miR-mut groups using the anti-YY1 or anti-H3K27Me3 antibodies respectively, and the products of the pull-down assay were specifically identified by PCR. The results showed that the amount of PCR product of the TET2 promoter fragment bound to YY1 in the miR-544 group was significantly less than that in the miR-mut group. Meanwhile, the amount of PCR product of the TET2 promoter fragment bound to H3K27Me3 in the miR-544 group was also significantly less than that in the miR-mut group (Fig. 5). The above experiments suggested that during the differentiation of HuAECs into iECICs, high expression of miR-544 can significantly reduce the negative regulatory effect of the PRC2 complex (in which YY1 is located) on the activation of TET2 transcription, resulting in increased transcription and expression of TET2.

### The differentiation of HuAECs into iECICs is accompanied by hydroxymethylation of genomic DNA

**Fig. 6:**
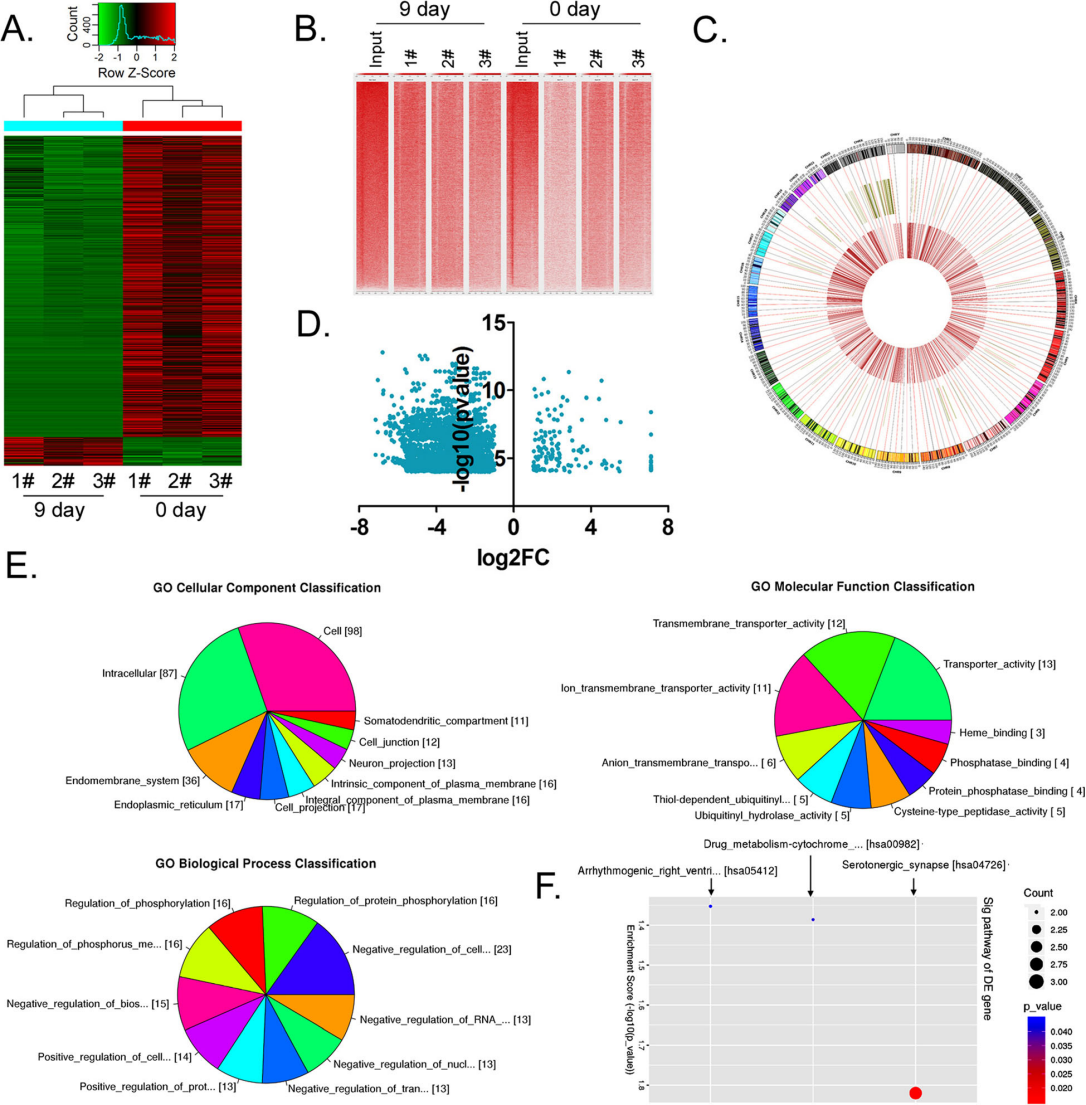
Hydroxymethylation of genomic DNA of iEClCs before and after directed differentiation by hMeDIP-sequencing. **a** Z-Score transformation heat map of differentially hydroxymethylated DNA sequences. **b** + 1 TSS & TES profiles of coding genes with differential hydroxymethylation status. **c** A Circos plot of different chromosomal locations where differentially-methylated/hydroxymethylated DNA sequences are located. **d** Statistical scatter plot of the distribution of differentially hydroxymethylated sequences. **e** The results of Gene Ontology analysis. **f** The results of KEGG Pathway analysis

As the aforementioned results have demonstrated, there was a significant change in the expression level of the DNA hydroxymethylase TET2 during the differentiation of HuAECs into iECICs, suggesting that there might be a change in the hydroxymethylation status of genomic DNA during cell differentiation. Therefore, we analysed the difference in genomic DNA hydroxymethylation between HuAECs (before induction) and iECICs (after induction) using hMeDIP-sequencing technology (Fig. 6). In the hMeDIP-sequencing assay, fragments of genomic DNA from HuAECs and iECICs showed 70,749,358 nucleic acid peaks and 64,014,296 nucleic acid peaks, respectively. The matching rate of samples was between 41 and 77%, which was consistent with normal distribution. After statistical analysis, we found that, compared with HuAECs, iECICs had 174 protein-coding genes with extensive hydroxymethylation modifications (log_2_FC > 1.0; *p*-value< 0.001) (Supplementary hMeDIP-Seq). The gene ontology (GO) analysis and the Kyoto Encyclopaedia of Genes and Genomes (KEGG) were used to further study the sequencing data. GO analysis is generally divided into three parts: molecular function (MF), biological process (BP), and cellular component (CC). The results indicated that genes with extensive hydroxymethylation were mainly involved inbiological processes, such as negative regulation of cellular metabolic processes, regulation of phosphorus metabolic processes, and negative regulation of biosynthetic processes. In the CC domain, these genes were mainly involved in the structural composition of the cell. In the MF domain, these genes were mainly involved indifferent functions, such as transmembrane transporter activity and transporter activity (Fig. 6, Supplementary hMeDIP-Seq). In addition, the aforementioned highly hydroxymethylated genes were studiedwith reference to the KEGG database (http://www.genome.jp/kegg/), and three signal transduction pathways (Serotonergic synapse, Drug metabolism-Cytochrome P450, Arrhythmogenic right ventricular cardiomyopathy) were found to be upregulated due to hydroxymethylation of the aforementioned genes (Fig. 6; Supplementary hMeDIP-Seq). The results showed that the differentiation of HuAECs into iECICs was accompanied by hydroxymethylation of genomic DNA.

### Overexpression of miR-544 promotes hydroxymethylation of genomic DNA of iECICs

**Fig. 7:**
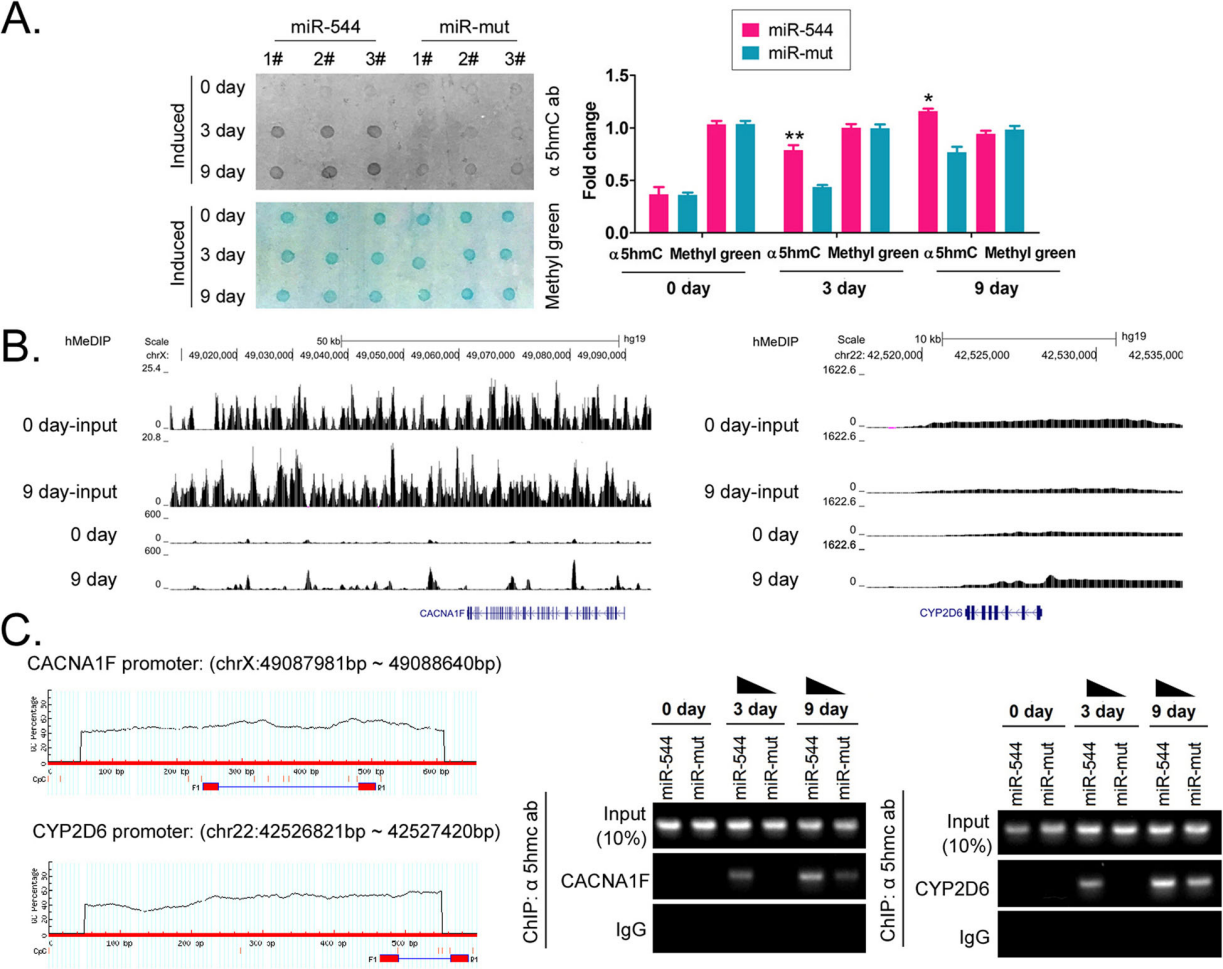
Detection of 5′-hydroxymethylation of specific sites of CACNA1F and CYP2D6 genes. **a** Dot plotting assay of the overall hydroxymethylation level of genomic DNA. **p < 0.01 vs. miR-mut; *p < 0.05 vs. miR-mut; t test; n = 3. **b** Visual analysis of hMeDIP-sequencingresults of modified sites of genes. **c** ChIP-PCR analysis of 5′-hydroxymethylation of specific sites of CACNA1F and CYP2D6 genes. F1 and R1 are primer sites used for PCR

Dot blotting results showed that the overall hydroxymethylation level of genomic DNA was significantly higher in the miR-544-iECICs group than in the miR-mut-iECICs control group during the initial stage of induction (day 3) (Fig. 7). The high level of hydroxymethylation of the aforesaid genomic DNA was maintained until the ninth day of differentiation induction (Fig. 7). Although, on the ninth day, the level of hydroxymethylation of genomic DNA in the miR-mut-iECICs group was slightly elevated, it was still significantly lower than that in the miR-544-iECICs group. The results suggested that the overexpression of miR-544 in HuAECs promoted the overall hydroxymethylation level of genomic DNA of iECICs by activating the expression of TET2.

Given the KEGG pathway prediction based on hMeDIP-sequencing results, the promoters or coding sequences of the specific genes (CACNA1F, CYP2D6, and MAOA) involved in serotonergic synapse pathway are highly hydroxymethylated (Fig. 7). The genomic DNA of each group of cells was enriched by anti-5-hmc antibody, which provided the template for the amplification of target gene sequences by specific primers. ChIP-PCR results showed that in the miR-544-iECICs group, specific sites in the promoter region of CACNA1F and CYP2D6 had undergone 5′-hydroxylation at the initial stage of induction (Day 3) (Fig. 7). At the same time, in the miR-544-iECICs group, the specific sites of the aforesaid genes did not undergo 5′-hydroxylation (no positive sequence cross-linked with the anti-5-hmc antibody was obtained in PCR) (Fig. 7). Although, by the 9th day of induction, positive bands were obtained in both groups in PCR targeting specific sites of CACNA1F and CYP2D6, the positive band of the miR-544-iECICs group was significantly brighter than that of the miR-544-iECICs group. The results suggested that overexpression of miR-544 could induce significant 5′-hydroxylation of specific regions of CACNA1F and CYP2D6 promoters in the early stage of directed differentiation. It also provided basic conditions for promoting the serotonergic synapse stimulating pathway.

### Overexpression of miR-544 promotes ability of maturation, antioxidation and vascular formation in vitro

**Fig. 8:**
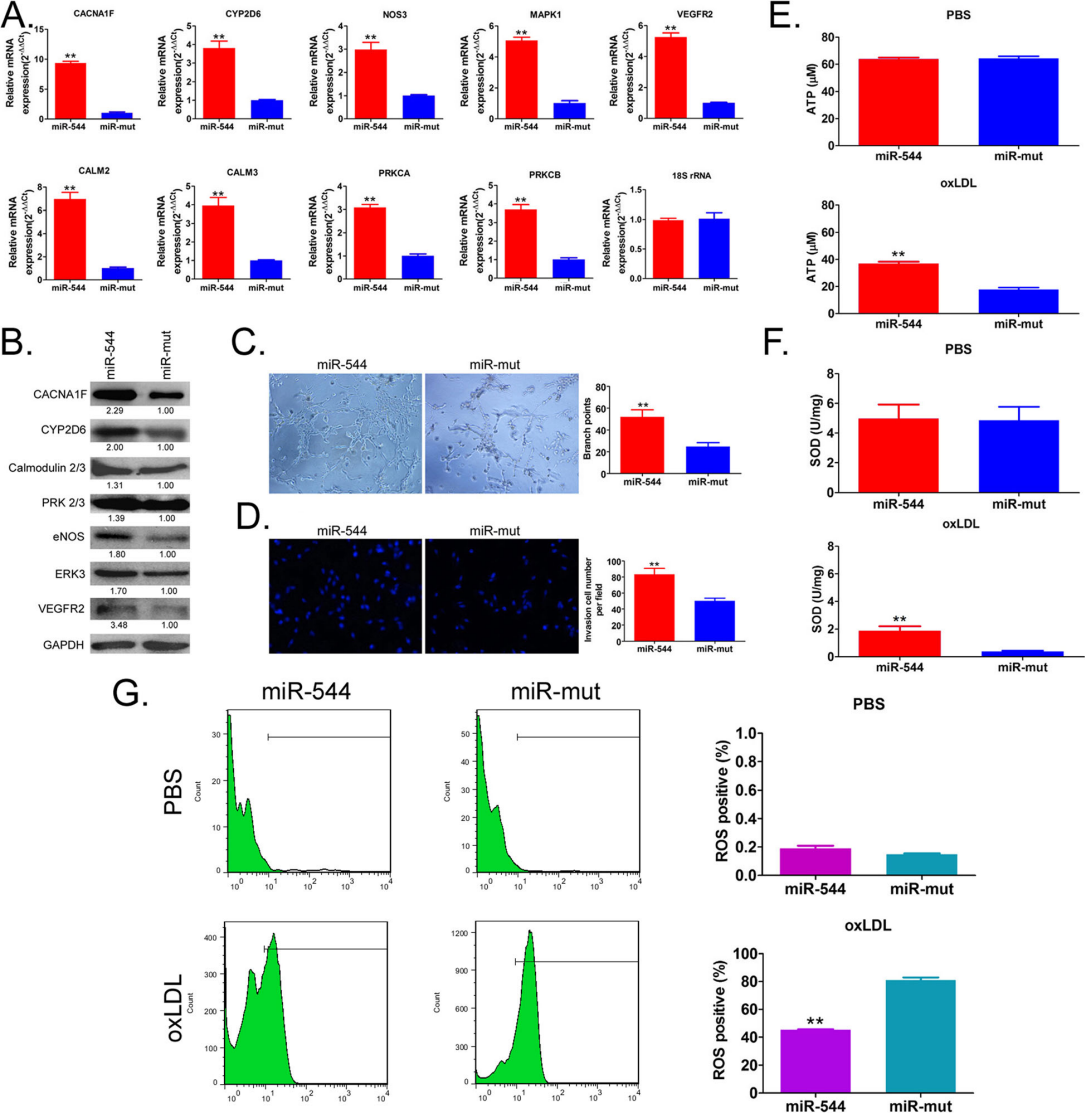
Overexpression of miR-544 promotes the function of iEClCs. **a** qPCR assay of the expression of mRNA of key factors involved in the serotonergic synapse stimulating pathway in each group of iEClCs. **p < 0.01 vs. miR-mut; t test; n = 3. **b** Western blot analysis of the expression of key factors in the serotonergic synapse stimulating pathway in each group of iEClCs. **c** Angiogenesis assay of each group of iEClCs in the extracellular matrix. **p < 0.01 vs. miR-mut; t test; n = 3. **d** Transwell assay. **p < 0.01 vs. miR-mut; t test; n = 3. **e** ATP levels in the iEClCs of each group. **p < 0.01 vs. miR-mut; t test; n = 3. **f** SOD levels in the iEClCs of each group. **p < 0.01 vs. miR-mut; t test; n = 3. **g** Flow cytometry showing the proportions of ROS-positive iEClCs of each group. **p < 0.01 vs. miR-mut; t test; n = 3

To evaluate the effect of overexpression of miR-544 on post-differentiation function of iECICs, we first examined the expression of key downstream factors in the serotonergic synapse pathway. The results of qPCR indicated that the expression levels of key factors, including CACNA1F, NOS3, CALM2, CAML3, PRKCA, MAPK1, CYP2D6, and VEGFR2, in the miR-544-iECICs group were significantly higher than those in the miR-mut-iECICs group (Fig. 8). Western blot analysis showed that the expression levels of CACNA1F, eNOS, Calmodulin 2/3, PRK 2/3, ERK3, CYP2D6, and VEGFR2 in the miR-544-iECICs group were significantly higher than those in the control group (Fig. 8). The results suggested that overexpression of miR-544 induced the serotonergic synapse pathway and promoted the differentiation and maturation of iECICs. Next, the results of transwell assay showed that the number of miR-544-iECICs that penetrated the extracellular matrix and migrated to the back of the membrane was significantly higher than that of miR-mut-iECICs (Fig. 8). Besides, the three-dimensional angiogenesis assay showed that miR-544-iECICs formed three-dimensional blood vessels in the extracellular matrix, and their number of nodes between the lumens was significantly higher than that of miR-mut-iECICs (Fig. 8). The ROS assay showed that the ROS level was lower in miR-544-iECICs than in miR-mut-iECICs (Fig. 8). However, the SOD and ATP levels were significantly higher in miR-544-iECICs than in miR-mut-iECICs (Fig. 8). The results suggested that overexpression of miR-544 enhanced the ability of maturation, antioxidation and vascular formation.

### Transplant of miR-544-iECICs effectively improves antioxidation and plaque deposits in atherosclerotic mice

**Fig. 9:**
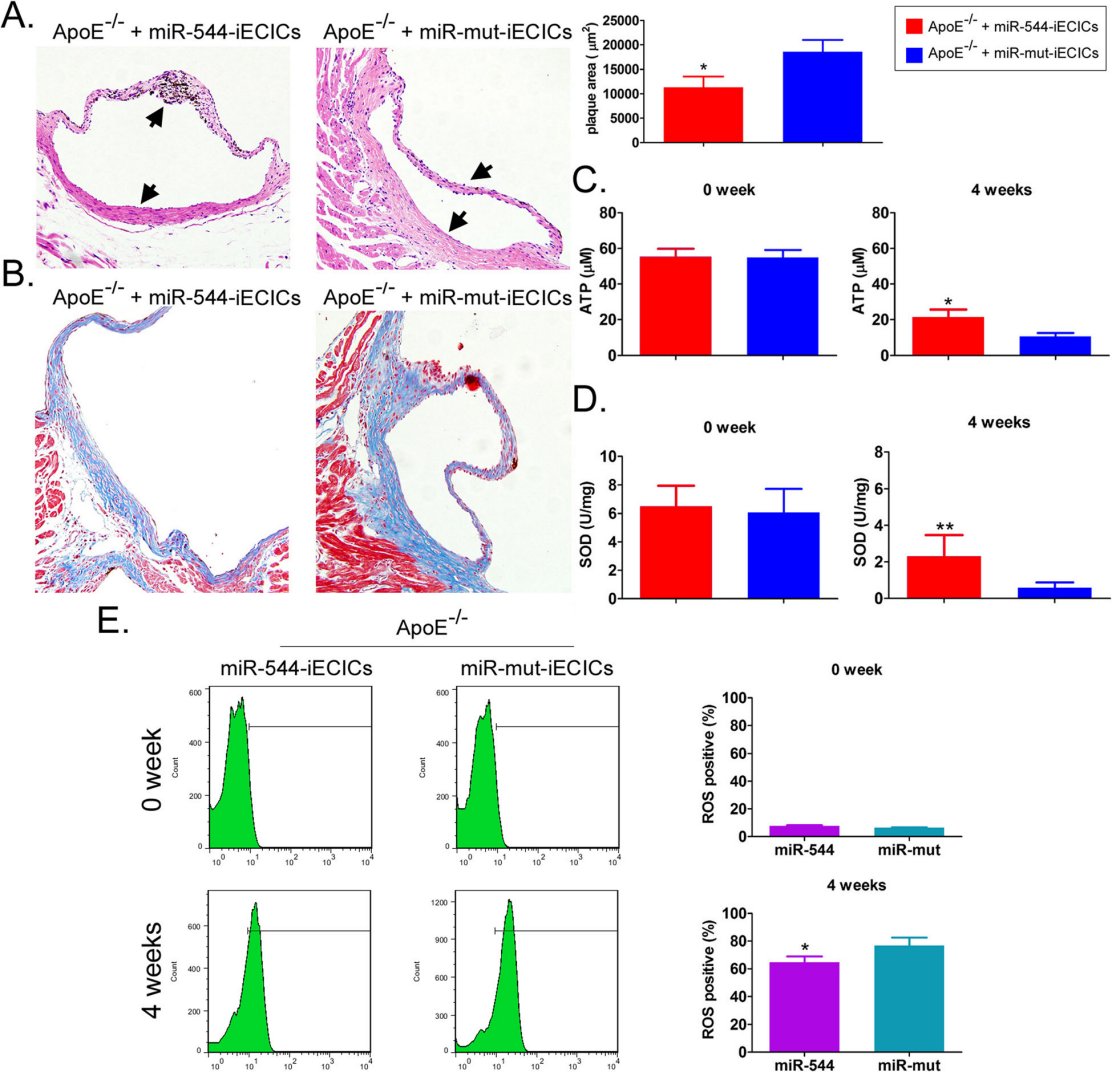
iECICs are effective in improving antioxidation and plaque deposits in ApoE^−/−^ and atherosclerotic mice model. **a** HE staining of tissues. Magnification: 200×. Plague area test. *p < 0.05 vs. ApoE^−/−^ mice transplanted with miR-mut-iECICs; t test; *n* = 6. **b** Masson staining of tissues. Magnification: 200×. **c** ATP levels in vascular endothelial cells of abdominal aorta of ApoE−/− mice transplanted with miR-544-iECICs or miR-mut-iECICs and fed with high-fat diet. *p < 0.05 vs. ApoE^−/−^ mice transplanted with miR-mut-iECICs; t test; n = 6. **d** SOD levels in vascular endothelial cells of abdominal aorta of ApoE−/− mice transplanted with miR-544-iECICs or miR-mut-iECICs and fed with high-fat diet. **p < 0.01 vs. ApoE^−/−^ mice transplanted with miR-mut-iECICs; t test; n = 6. **e** Flow cytometry showing the proportions of ROS-positive vascular endothelial cells of abdominal aorta of ApoE−/− mice transplanted with miR-544-iECICs or miR-mut-iECICs and fed with high-fat diet. *p < 0.05 vs. ApoE^−/−^ mice transplanted with miR-mut-iECICs; t test

To examine whether iECICs of each group could prevent the occurrence of atherosclerosis, miR-544-iECICs and miR-mut-iECICs were transplanted into ApoE^−/−^ mice fed with high-fat diet. The pathological results of the cardiovascular tissues of mice showed no significant thickening, lipid deposition, or plaque deposition in the aortic arch, regardless of the type of iECICs transplanted (Fig. 9). Besides, the vascular endothelial cells were intact and no significant oedema, inflammation, or necrosis was observed (Fig. 9). The Masson trichrome staining suggested that although there was a small amount of lipid infiltration in the aortic arch of mice, the collagen layer was intact without signs of thickening in both groups (Fig. 9). Occasionally, a small amount of lipid infiltration was observed in the collagen layer of the vascular interstitium (Fig. 9). The results of the ROS assay showed that the ROS level of abdominal aorta was lower in miR-544-iECICs tranplant group than in miR-mut-iECICs tranplant group (Fig. 9). However, the SOD and ATP levels were significantly higher in miR-544-iECICs tranplant group than in miR-mut-iECICs tranplant group (Fig. 9). These results suggested that the tranplant of miR-544-iECICs significantly reduced oxidative stress injury in ApoE^−/−^ atherosclerotic mice.

## Discussion

Amniotic epithelial cells are more abundant than epithelial cells, and they are as pluripotent as embryonic stem cells [11–13]. Such characteristics make them potentially useful in clinical treatment. There have been many reports that amniotic epithelial cells can differentiate into various adult cells derived from the three germ layers in vitro and in vivo using specific induction methods [11–13]. Adult cells derived from amniotic epithelial cells are very similar to typical adult cells in terms of both phenotype and function [11–13]. Based on the aforesaid clues, we hypothesized that it is possible to direct the differentiation of amniotic epithelial cells into endothelial progenitor cells and endothelial cell-like cells in vitro using specific methods. Based on previous studies oninducingthe differentiation of iPS cells into endothelial progenitor cells and endothelial-like cells [36, 37, 41], we found that the same methods could be used to inducethe differentiation of amniotic epithelial cells into endothelial-like cells. Such endothelial-like cells express high levels of markers of endothelial progenitor cell (CD34+, CD146^+^, VE-cadherin^+^, ETV2^+^, VEGFR2^low^, etc.) and also partially expressedmarkers of mature vascular endothelial cells (CD34^low^, CD146^low/−^, VE-cadherin^+^, ETV2^low^, VEGFR2^+^, etc.). However, since the cells obtained from induction are not uniform, it has been suggested that during the differentiation process, different subpopulations of cells are produced, and there are various states of differentiation and maturation of cells. We examined the aforementioned phenomena from the perspective of epigenetics. The process of differentiation of stem cells into adult cells is accompanied with dramatic phenotypic changes, and there are different regulationsof certain genes at the transcriptional and post-transcriptional level, whichare often closely related to different types of epigenetic regulation [24–28]. This study focused on a well-known epigenetic regulator, Yin yang-1 (YY1).YY1 was first cloned and defined by Park and Shi et al. in 1991 [32]. It is widely expressed in a variety of tissues and is involved in numerous biological processes, such as chromatin remodelling, cellular proliferation/differentiation/apoptosis, and embryogenesis [29–32]. YY1 exerts its effect by regulation of target genes. As a transcription factor, YY1 not only activates the transcription of target genes, but also inhibits the transcriptional activity of specific target genes [29–32]. Moreover, as a cofactor or scaffold protein, YY1 can function by interacting with other proteins [29–32]. Through experiments and bioinformatics prediction, we discovered that miR-544 potentially regulates YY1. When amniotic epithelial cells overexpressing miR-544 are induced to undergo directed differentiation, the cells express a large number of markers of mature vascular endothelial cells. This result suggested that YY1 gene is an important factor in maintaining the pluripotency of stem cells. In the presence of YY1 protein, although the stem cells can be induced to transform into target adult cells, most of the cells remain progenitor cells that are not fully differentiated and mature. When the expression of YY1 is silenced, most of the stem cells can differentiate into mature adult cells.

Subsequently, we explored the in-depth mechanism of the aforementioned findings. Considering YY1 is a polycomb group (PcG) protein, it can induce activation or inhibition of transcription of downstream genes. Besides, the differentiation of stem cells is accompanied by hydroxymethylation of genomic DNA. Therefore, we analysed the expression of the DNA hydroxymethylase family proteins (TET). The results confirmed that there was a significant difference in the expression level of TET2 before and after the differentiation of amniotic stem cells. While the overexpression of miR-544 silenced the expression of endogenous YY1, the expression of TET2 increased. Subsequently, it was confirmed by epigenetic experiments that the transcriptional activity of TET2 was regulated by PRC2 of the PcG family. When the expression of YY1 was silenced by miR-544, it was unable to recruit EZH2, another key factor in PRC2, thus failing to induce a 3′-methylation of histone H3K27. The unmodified H3K27 fails to bind to promoter of TET2, activating the transcription and expression of TET2.

Then, after TET2 is expressed, what other epigenetic events occurred? hMeDIP-sequencing revealed that there were significant hydroxymethylation modifications of the promoters or coding sequences of certain genes after induction. Moreover, through bioinformatics analysis, it was found that there were traces of hydroxymethylation modifications in the promoters or coding sequences of the key factors of the serotonergic synapse pathway, including CACNA1F, CYP2D6, and MAOA. Further studies have found that, after overexpression of miR-544, amniotic epithelial cells undergo large-scale hydroxymethylation modification of genomic DNA during the early stage of induced differentiation (around day 3). Some key factors of the serotonergic synapse stimulating pathway are also significantly activated and expressed. Previous studies have found that vascular endothelial cells form the wall lining of blood vessels, forming a smooth surface for blood flow. Vascular endothelial cells and the basement membrane form a permeability barrier through which liquids, gases, and macromolecules can selectively pass [42–44]. Peripheral serotonin is mainly found in vascular endothelial cells and platelets [42–44]. Serotonin, histamine, and bradykinin stimulate contraction of microfilaments and alter the width of the intercellular space and the tightness of cellular junctions, affecting and regulating vascular permeability [42–44]. Besides, angiotensin-converting enzyme can be found on the surface of vascular endothelial cells, which converts angiotensin I in plasma into angiotensin II, causing blood vessels to contract [42–44]. Vascular endothelial cells can also degrade serotonin, histamine, and norepinephrine. This indicates a close relationship between serotonin and vascular endothelial cells [42–44]. This intrinsic relationship is evident during the differentiation of amniotic epithelial cells into vascular endothelial-like cells. Overexpression of miR-544 significantly inhibits the expression of YY1, resulting in the activation of TET2 transcription and serotonin synaptic stimulating pathway, which promotes the differentiation and maturation of endothelial-like cells. Besides, both transwell assay and angiogenesis assay revealed that the endothelial-like cells with miR-544 overexpression show stronger physiological and biochemical activities.

**Fig. 10:**
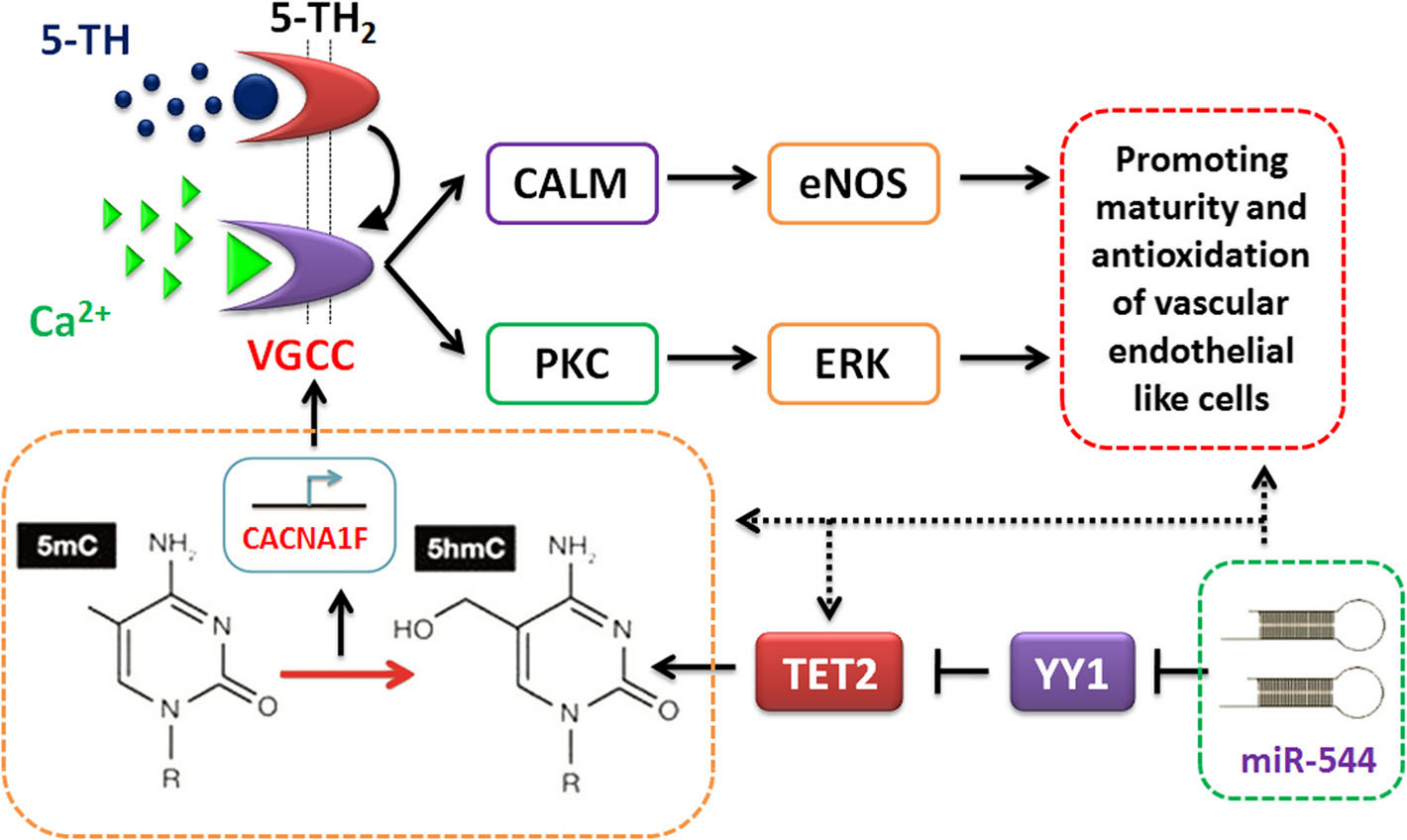
The molecular mechanism of miR-544 regulating the maturity and antioxidation of HuAECs-derived iECICs by regulating the YY1/TET2/serotonergic synapse signalling axis

Therefore, the entire study demonstrated that the process of induced differentiation of amniotic epithelial cells into vascular endothelial-like cells is accompanied by changes in multiple epigenetic events, and the entire regulatory process involves multiple steps at the transcriptional and post-transcriptional levels, including DNA demethylation (hydroxymethylation), covalent modification of histones, and microRNA-induced modification at the post-transcriptional level. Several alterations in epigenetic regulation ultimately activate a very important neurotransmitter pathway, the serotonergic synapse stimulating pathway. Activation of this pathway ultimately gives rise to more mature and functional vascular endothelial cells (Fig. 10). In this study, although we found that miR-544/YY1/TET2 axis regulated the maturity and antioxidation of iECICs derived from HuAECs, the mechanism of iECICs to alleviate atherosclerotic symptoms and antioxidation remains to be further studied. At the same time, the iECICs release of exosomes and nutrients can not be ignored in the process of restoration. We think our study has the possibility of further expansion.

## Conclusions

We proved that miR-544 regulates the maturity and antioxidation of iECICs derived from HuAECs by regulating the YY1/TET2/serotonergic synapse signalling axis. Meanwhile, our study also confirmed that the serotonergic synapse stimulating pathway is a key pathway involved in differentiation of stem cells into vascular endothelial cells and has a potential regulatory effect on the differentiation, maturation and antioxidation of vascular endothelial cells. And, our research has fully proved the advantage of HuAECs-derived iECICs in alleviating the symptoms of atherosclerosis, and provided the novel insights for the clinical translational medicine application of this cell.

## Supplementary information

**Additional file 1.** The results of hMeDIP-Seq assay.

**Additional file 2.**The full materials and methods in this study.

## Data Availability

The datasets used and/or analysed during the current study are available from the corresponding author on reasonable request.

## Abbreviations

5-caC: 5-carboxycytosine
5-caC: 5-carboxylcytosine
5-fC: 5-formylcytosine
5-hmC: 5-hydroxymethylcytosine
BP: Biological process
CC: Cellular component
Cdh5: VE-Cadherin
co-IP: co-immunoprecipitation
ECs: Endothelial cells
FCM: Flow cytometry
GO: Gene ontology
hMeDIP-Seq: Human methylated DNA immunoprecipitation sequencing
HuAECs: Human amniotic epithelial cells
iEClCs: Induced endothelial cell-like cells
IF: Immunofluorescence
iPS: Induced pluripotent stem
KEGG: Kyoto Encyclopaedia of Genes and Genomes
MF: Molecular function
PRC2: Polycomb repressive complex 2
TDG: Thymidine-DNA glycosylase
TET2: Ten-eleven translocation 2
YY1/Yy1: YinYang 1
