# Supplementary material for: miR-544 promotes maturity and antioxidation of stem cell-derived endothelial like cells by regulating the YY1/TET2 signalling axis
Source: Cell Commun Signal. 2020 Mar 3;18:35. doi: 10.1186/s12964-019-0504-6 (PMC7055126; doi:10.1186/s12964-019-0504-6)
Supplement: Supplementary file 3 — Additional file 2.The full materials and methods in this study. [file 12964_2019_504_MOESM3_ESM.zip › Table S1.docx]

**Table S1 The antibodies**

| **Antibodies** | **Cat. Number** | **Companies** | **Applications** |
| --- | --- | --- | --- |
| Rabbit anti-human Oct4 antibody [EPR17929] | # ab181557 | Abcam | IF (1:200)  WB (1:1000) |
| Rabbit anti-mouse SSEA4 antibody [MC813] | # ab16287 | Abcam | IF (1:200)  WB (1:1000) |
| Rabbit anti-human VE Cadherin antibody [3D5C7] | # ab166715 | Abcam | IF (1:200)  WB (1:1000) |
| Rabbit anti-human CD34 antibody [EP373Y] | # ab81289 | Abcam | IF (1:200)  WB (1:1000) |
| Rabbit anti-human VEGF Receptor 2 antibody | # ab2349 | Abcam | IF (1:200)  WB (1:1000) |
| Rabbit anti-human YY1 [H-10] antibody | # sc-7341 | Santa Cruz Biotechnology | IF (1:200)  WB (1:1000) |
| Rabbit anti-human Tet2 antibody | # ab124297 | Abcam | ChIP (1:100)  WB (1:1000) |
| Rabbit anti-human KMT6 / EZH2 antibody | # ab186006 | Abcam | ChIP (1:100)  WB (1:1000) |
| Rabbit anti-human Histone H3 (tri methyl K4) antibody | # ab8580 | Abcam | ChIP (1:100)  WB (1:1000) |
| Rabbit anti-human 5-hydroxymethylcytosine (5-hmC) antibody [RM236] | # ab214728 | Abcam | ChIP (1:100)  WB (1:1000) |
| Rabbit anti-human CACNA1F antibody [EPR11822] | # ab171968 | Abcam | WB (1:1000) |
| Rabbit anti-human Cytochrome P450 2D6 antibody | # ab62204 | Abcam | WB (1:1000) |
| Rabbit anti-human Calmodulin 1/2/3 antibody [2D1] | # ab2860 | Abcam | WB (1:1000) |
| Rabbit anti-human PRK3+PRK2+PRK1 antibody [EPR1044] | # ab187660 | Abcam | WB (1:1000) |
| Rabbit anti-human eNOS antibody [M221] | # ab76198 | Abcam | WB (1:1000) |
| Rabbit anti-huamn GAPDH antibody [EPR16891] | # ab181602 | Abcam | WB (1:1000) |
| Goat anti-Rabbit IgG H&L (HRP) | # ab97051 | Abcam | WB (1:1000) |
| Goat anti-Mouse IgG H&L (HRP) | # ab6789 | Abcam | WB (1:1000) |
| Goat anti-Rabbit IgG H&L (Alexa Fluor® 488) | # ab150077 | Abcam | IF (1:200) |
| Goat Anti-Mouse IgG H&L (Cy3) preadsorbed | # ab97035 | Abcam | IF (1:200) |
