# Supplementary material for: miR-544 promotes maturity and antioxidation of stem cell-derived endothelial like cells by regulating the YY1/TET2 signalling axis
Source: Cell Commun Signal. 2020 Mar 3;18:35. doi: 10.1186/s12964-019-0504-6 (PMC7055126; doi:10.1186/s12964-019-0504-6)
Supplement: Supplementary file 3 — Additional file 2.The full materials and methods in this study. [file 12964_2019_504_MOESM3_ESM.zip › Table S2.docx]

**Table S2 The primers**

| **Gene Name** | **Forward primers (5'→3')** | **Reversed primers (5'→3')** |
| --- | --- | --- |
| Yy1 | AAGAGCGGCAAGAAGAGTTAC | CAACCACTGTCTCATGGTCAATA |
| Oct4 | CAGTGCCCGAAACCCACAC | GGAGACCCAGCAGCCTCAAA |
| Cdh5 | GTTCACGCATCGGTTGTTCAA | CGCTTCCACCACGATCTCATA |
| miR-186 | CAAAGAATTCTCCTTTTGGGCT | GCTGTCAACGATACGCTACCTA |
| miR-544 | ATTCTGCATTTTTAGCAAGTTC | GCTGTCAACGATACGCTACCTA |
| miR-433 | ATCATGATGGGCTCCTCGGTGT | GCTGTCAACGATACGCTACCTA |
| miR-124 | TAAGGCACGCGGTGAATGCCAA | GCTGTCAACGATACGCTACCTA |
| miR-506 | TAAGGCACCCTTCTGAGTAGA | GCTGTCAACGATACGCTACCTA |
| miR-152 | AGGTTCTGTGATACACTCCGACT | GCTGTCAACGATACGCTACCTA |
| miR-192 | CTGACCTATGAATTGACAGCC | GCTGTCAACGATACGCTACCTA |
| miR-215 | TCTGTCATTTCTTTAGGCCAATA | GCTGTCAACGATACGCTACCTA |
| CACNA1F promoter | GGAGTTTTGTTTTTGTTGTTTAGGT | ACTCATACCTATAATCCCAACACTTTC |
| CYP2D6 promoter | TTAGGTAGTAAGAGGTGAAGTTGTTA | CTCCCAAAATACTAAAATTACAAAC |
| CACNA1F | AAGGCATTGAGGGCGTTTC | GCATTCGTCCAAGGAACAGC |
| CYP2D6 | TGGCAAGGTCCTACGCTTC | GCCACCACTATGCACAGGTT |
| NOS3 | TGATGGCGAAGCGAGTGAAG | ACTCATCCATACACAGGACCC |
| CALM3 | GACCATTGACTTCCCGGAGTT | GATGTAGCCATTCCCATCCTTG |
| PRKCA | ATGTCACAGTACGAGATGCAAAA | GCTTTCATTCTTGGGATCAGGAA |
| PRKCB | AAACCTTGTACCTATGGACCCC | CCCAATCCCAAATCTCTACTGAC |
| MAPK1 | TCTGGAGCAGTATTACGACCC | CTGGCTGGAATCTAGCAGTCT |
| VEGFR2 | GTGATCGGAAATGACACTGGAG | CATGTTGGTCACTAACAGAAGCA |
| 18S rRNA | CAGCCACCCGAGATTGAGCA | TAGTAGCGACGGGCGGTGTG |
